# A brief review: the therapeutic potential of bone marrow mesenchymal stem cells in myocardial infarction

**DOI:** 10.1186/s13287-017-0697-9

**Published:** 2017-11-02

**Authors:** Chi Miao, Mingming Lei, Weina Hu, Shuo Han, Qi Wang

**Affiliations:** Cardiovascular Department Of Internal Medicine, The Fourth Affiliated Hospital of China Medical University, Chongshandong Street No.4, Shenyang, 110032 China

**Keywords:** Myocardial infarction, Bone marrow mesenchymal stem cells, Regeneration, Transplantation

## Abstract

Myocardial infarction (MI) results in dysfunction and irreversible loss of cardiomyocytes and is among the most serious health threats today. Bone marrow mesenchymal stem cells (BMSCs), with their capacity for multidirectional differentiation, low immunogenicity, and high portability, can serve as ideal seed cells in cardiovascular disease therapy. In this review, we examine recent literature concerning the application of BMSCs for the treatment of MI and consider the following aspects: activity of transplanted cells, migration and homing of BMSCs, immunomodulatory and anti-inflammatory effects of BMSCs, anti-fibrotic activity of BMSCs, the role of BMSCs in angiogenesis, and differentiation of BMSCs into cardiomyocyte-like cells and endothelial cells. Each aspect is complementary to the others and together they promote the repair of cardiomyocytes by BMSCs after MI. Although transplantation of BMSCs has enabled new options for MI treatment, the critical issue we must now address is the reduced viability of transplanted BMSCs due to inadequate blood supply, poor nourishment of cells, and generation of free radicals. More clinical trials are needed to prove the therapeutic potential of BMSCs in MI.

## Background

Cardiovascular disease is a major problem harmful to human health worldwide due to its high incidence. In the event of acute myocardial infarction (MI) or other heart diseases, the number of surviving cardiocytes declines. With the formation of fibrous cicatricial tissue and remodeling of ventricular tissue after MI, the contraction ability of the myocardium declines. Eventually, the decline in cardiac function may induce the reoccurrence of heart failure as a further threat to life. Although thrombolysis, coronary implantation of stents, and coronary artery bypass grafting have been used to treat MI clinically, these methods cannot reverse the damage to infarcted myocardium or improve heart function fundamentally. Finding a method to improve cardiac function through regeneration of damaged and nonfunctioning myocardium has therefore become a new direction for research. In recent decades, scientists have exerted great effort and energy into the use of stem cells to repair damaged myocardium and restore cardiac function. Currently, transplantation of exogenous stem cells into infarcted myocardium is regarded as a promising route for treating MI. Bone marrow mesenchymal stem cells (BMSCs) have emerged as the primary research focus among all of the various types of stem cells examined. A recent study has shown that pharmacological activation of the exchange protein directly activated by the cAMP (Epac)/Rap1 signaling pathway improved cardiac function through better survival, adhesion, and differentiation of transplanted BMSCs in rat [1]. A further study demonstrated that BMSCs preconditioned with 2,4-dinitrophenol improved cardiac function after transplantation, which was attributed to the improvement of survival, homing, and adhesion, as well as cardiomyogenic and angiogenic differentiation of these cells in vivo [2]. The application of BMSCs in MI has entered a phase of clinical research. Rodrigo et al. [3] found that intramyocardial injection of autologous BMSCs in acute MI patients is feasible and safe after 5 years of follow-up. Another research study showed that intracoronary infusion of human BMSCs at 1 month is tolerable and safe with modest improvement in left ventricular ejection fraction at 6-month follow-up [4]. Even allogeneic BMSCs were used in clinical trials, and found safe and well tolerated when administered intravenously in acute MI patients 2 days after percutaneous coronary intervention up to 2 years of follow-up [5].

## General conditions of MI

MI is a disease that seriously threatens people’s health. Degeneration of cardiac tissues, a major cause of mortality in the western world, is likely to become a greater problem in the forthcoming decades. Cardiac damage is associated with dysfunction and irreversible loss of cardiomyocytes [6]. At present, drugs, intervention, and coronary artery bypass grafting treatment are used to partially improve myocardial ischemia, but these methods cannot rescue tissue damaged by MI. The main reasons for the increased incidence of heart failure and mortality after MI are the local reduction in the number of cardiocytes in the infarcted area caused by high oxidative stress in the inflammatory microenvironment, as well as tissue fibrosis, formation of scar tissue, and cardiac remodeling. Consequently, clinicians have begun to seek a new method for the treatment of heart failure after MI.

## Characteristics of mesenchymal stem cells

Mesenchymal stem cells (MSCs), originating from mesoderm adult stem cells, retain the ability to amplify and self-renew in vitro and differentiate into cartilage, fat, bone, and nerve cells under certain conditions. MSCs are in great demand because of their low immunogenicity and immunomodulatory capacity, effectively reducing or avoiding immune rejection. MSCs can be derived from a variety of tissues including bone marrow, fat, muscle, and synovial membrane. Among all MSCs examined, BMSCs are the most popular in application because of their obvious advantages. In addition to their relatively easy acquisition from an extensively rich resource, BMSCs are free from ethical issues because they can be transplanted autologously. Furthermore, the relatively simple cultivation and separation, rapid proliferation, and high genetic stability of BMSCs allow for their easy amplification in vitro. Finally, their capacity for multidirectional differentiation, low immunogenicity, and high portability make BMSCs ideal seed cells for therapy of cardiovascular diseases. Because of the activity of chemotactic protein stromal cell derived factor 1 (SDF-1) and its receptor CXC chemokine receptor 4 (CXCR4), BMSCs have the ability for automatic homing on the infarction area after transplantation [7–9], significantly facilitating the treatment of cardiovascular disease. The therapeutic effect of BMSCs involves a series of mechanisms after their transplantation into ischemic myocardium. These include the differentiation and fusion of BMSCs to cardiocytes and endothelial cells; paracrine effects related to the secretion of various cytokines like vascular endothelial growth factor (VEGF), interleukin (IL)-6, platelet-derived growth factor, fibroblast growth factor (FGF), hepatocyte growth factor (HGF), SDF-1, and insulin-like growth factor (IGF), which promote the restoration of cardiac function; the mobilization of autologous cardiac stem cells or progenitor cells to differentiate into cardiomyocytes and proliferation under the microenvironment created by BMSCs to continuously improve cardiac function; and the inhibition of inflammatory responses by reducing inflammatory cytokine levels and gene expression to protect the myocardium. Many studies [10, 11] have shown that transplantation of BMSCs after MI greatly promotes the differentiation of cardiocytes surrounding the infarction area, enhances angiogenesis, increases apoptotic resistance, and exerts anti-fibrotic effects, significantly improving myocardial repair after MI. Combined shock wave and autologous BMSC therapy has also been shown to be more effective than either therapy alone in inhibiting apoptosis, inflammation, and oxidative stress, as well as in augmenting angiogenesis in the infarcted area in animal models [12].

## Activity of transplanted cells

After MI, the ischemic anoxic environment seriously affects the survival of transplanted cells. In-depth research into BMSC transplantation in the treatment of MI has led to the realization that the effect of single-cell transplantation is limited. Therefore, researchers have tried to improve the harsh environment after infarction and enhance the capacity of transplanted cells to resist ischemic anoxia. Numerous studies discussed in the following have reported that hypoxic preconditions, various drug combinations, and joint cytokine and gene transfection treatments can improve the anti-oxidative stress capacity of transplanted BMSCs and increase their survival rate.

As a novel strategy for preconditioning BMSCs, treatment with hypoxia-inducible factor (HIF)-1α and the prolyl hydroxylase inhibitor dimethyloxalylglycine has been shown to greatly improve the cell survival rate in vivo and in vitro, and to reduce the area of MI to enhance the effect of cell therapy [13]. Some researchers have suggested that the modification of BMSCs with a fused gene expressing fibroblast growth factor 4 and basic fibroblast growth factor (bFGF) can not only increase the expression of bFGF but also enhance the survival of the transplanted cells and improve cardiac functions in animal models [14]. Mao et al. [15] found that microRNA (miR)-23a inhibited apoptosis of BMSCs induced by TNF-α through the regulation of caspase-7 in vitro, and that injection of BMSCs overexpressing miR-23a reduced infarct size and improved left ventricular (LV) function in a rat MI model, providing an alternative treatment strategy for patients with heart failure caused by MI who are not optimal candidates for surgical treatment. The hypoxic microenvironment after MI can also affect the activity of BMSCs. Recent in-vitro research has suggested that apoptosis of BMSCs under hypoxic conditions is regulated by autophagy via the AMP-activated protein kinase/mammalian target of rapamycin pathway [16]. Another study showed that inhibition of inositol hexakisphosphate kinases has the potential to enhance functional survival of engrafted BMSCs via activation of the Akt signaling pathway in vitro [17]. Other researchers have found that treatment with a low dose of atorvastatin facilitates the survival of engrafted BMSCs and improves tissue repair, regeneration, and cardiac function after BMSC transplantation in an experimental animal model [18]. Wang et al. [19] found that the combination of BMSCs with melatonin pretreatment led to an effective reduction in intracellular reactive oxygen, which increased the levels of Bcl-2/Bax, decreased mitochondrial membrane potential, and activated caspase-3, thus promoting resistance to cell death induced under hypoxic and serum-free conditions, which may improve cardiac function in vivo.

## Migration and homing of BMSCs

In addition to cell viability, migration and homing of BMSCs is another crucial factor that influences their therapeutic effect. BMSCs demonstrate a migratory tendency to infarcted myocardial tissues after transplantation through the intravenous route, which can obviously delay deterioration in cardiac function caused by LV remodeling. However, because of the need to reach the infarction area by blood circulation, this method requires an excessive quantity of injected cells, which may cause venous embolism in a rat model [20]. Therefore, researchers have begun to explore methods for enhancing the migration and homing ability of BMSCs. Hu et al. [21] compared BMSCs pretreated with normal oxygen or 0.5% O_2_ for 24 h and found that the migration and homing of hypoxia-treated cells to the site of myocardial injury was enhanced significantly, which may be connected to Kv2.1 channel and focal adhesion kinase activation in vitro. Cheng et al. [22] demonstrated that Src family kinases were activated by SDF-1/CXCR4 signaling and played an essential role in the SDF-1/CXCR4-mediated BMSC chemotactic response and ischemic cardiac recruitment in a mouse model.

## Immunomodulatory and anti-inflammatory effects of BMSCs

MSCs possess the features of weak immunogenicity and antigen-presenting ability as a consequence of low expression of major histocompatibility complex (MHC) class II molecules and Fas ligand and no expression of MHC class I molecules, which enables MSCs to inhibit T-cell proliferation [23], resulting in immune exemption or immune tolerance. The immunomodulatory mechanism of MSCs mainly involves two aspects: inhibition of active inflammatory substances through a paracrine mechanism [24]; and the regulation of immune cells [25]. Guo et al. [26] found that expression of TNF-α, IL-1β, and IL-6 and cardiocyte apoptosis were markedly reduced after MSC transplantation, resulting in significant improvement of myocardial function in a mouse model. Ohnishi et al. [27] discovered that myocardial tissue levels of monocyte chemotactic protein-1 were significantly decreased after MSC transplantation in a rat model of acute myocarditis, and, accordingly, inflammatory cell infiltration was reduced. These studies suggest that MSCs are involved in immune regulation through paracrine mechanisms, and additional studies support their immunomodulatory influence on some immune cells. For example, the proliferation of T cells [28] and natural killer cells [29] and the maturation of dendritic cells [30] were inhibited by MSCs. The prostaglandin E-2 (PGE-2) receptor of macrophages was reported to interact with the PGE-2 receptor of MSCs, prompting M2 macrophages to release anti-inflammatory cytokine IL-10 and thus inhibit the inflammatory response in vitro [31]. B-cell amplification and the production of IgM, IgG, and IgA were restrained by soluble molecules produced by MSCs, resulting in the prevention of B-cell differentiation in vitro and in vivo [32].

As a type of MSC, BMSCs can also suppress the inflammatory response after MI. A study demonstrated that exosomes were the active ingredient of paracrine secretion by BMSCs, which stimulated neovascularization, restrained the inflammatory response, and improved cardiac function after ischemic injury in vitro and in vivo [33]. Hsu et al. [34] found that the CXCR4 antagonist TG-0054 improved impaired LV contractility following MI by mobilizing BMSCs to attenuate postinfarction inflammation in a porcine model.

## Anti-fibrotic effects of BMSCs

After the occurrence of MI, many cardiocytes die and are replaced by fibroblasts in the infarcted area. Heart failure is triggered by LV remodeling and arrhythmia, which is a major cause of cardiovascular death. Stem cells can inhibit the activation of fibroblasts, reduce the deposition of collagen and other extracellular matrix, thereby reducing LV remodeling, and improve cardiac function after MI mainly by regulating matrix metalloproteinases and inhibitors of matrix metalloproteinases. HGF, a well-known anti-fibrosis factor, was demonstrated to be a major contributor to the anti-fibrosis function of MSCs in vitro [35]. Another study revealed that BMSCs transplanted into the surrounding area after MI released HGF, which inhibited miR-155-mediated profibrosis signaling, thereby preventing cardiac fibrosis in a mouse model [36]. A novel study showed that nuclear factor (erythroid-derived 2)-like 2 (Nrf2) siRNA could effectively interfere with Nrf2 expression in BMSCs and reduce the repair ability of exogenous BMSCs in MI heart, which increased collagen deposition in the infarcted area, thus contributing to ventricular remodeling and reducing cardiac function in a rat model [37].

## The role of BMSCs in angiogenesis

Angiogenesis, an important process in the treatment of MI, refers to development of great vessels and capillaries. Abundant paracrine factors such as VEGF and SDF-1 are released by MSCs transplanted into the surrounding infarcted area, can regulate angiogenesis, and inhibit signaling pathways to promote the formation of novel vasoganglion.

An increasing volume of literature has reported that miRNAs participate in the regulation of vascular cell differentiation, proliferation, migration, and other biological processes. Some miRNAs possess the ability to promote angiogenesis, such as miR-126, miR-132, and others. The excessive expression of miR-126 facilitated angiogenesis around the infarcted area by regulating the AKT/ERK signaling pathway in a rat model [38]. Another study also reported that miR-126 promoted angiogenesis of MSCs via the AKT/ERK-related pathway in a mouse model [39]. Tang et al. [40] used a rat model of MI to compare the recovery after transplantation of BMSCs alone, treatment with VEGF alone, or VEGF treatment combined with BMSC transplantation. Combination therapy significantly increased vascular density, decreased the formation of scar tissue, and improved cardiac function compared to either treatment alone or to untreated infarcted control rats [40]. VEGF treatment also promoted the differentiation of CXCR4-positive BMSCs into vessel endothelial cells in vitro [41]. Another research study has suggested that overexpression of the cellular repressor of adenovirus E1A-stimulated genes in BMSCs may promote VEGF-induced angiogenesis by inhibiting Von Hippel-Lindau tumor suppressor-mediated HIF-1α degradation, consequently protecting against rat MI [42].

## Differentiation of BMSCs into cardiomyocyte-like cells and endothelial cells

MSCs present a zonal distribution in myocardial tissue after transplantation similar to the distribution of cardiocytes. The increase in cardiac-specific marker proteins such as troponin preliminarily confirms the differentiation of BMSCs into cardiocytes. The activation of Notch1 signaling contributed to multilineage differentiation of c-Kit^POS^/NKX2.5^POS^ BMSCs isolated from Sprague–Dawley rat femurs into cardiomyocytes [43]. Retrograde coronary venous infusion of bFGF further augments engraftment and differentiation of transplanted BMSCs, allowing for recovery of cardiac function and preventing adverse remodeling in a canine infarct model [44]. A combination of bFGF and hydrocortisone also promotes the differentiation of BMSCs into cardiomyocyte-like cells in vitro [45]. In addition, overexpression of miRNA-1-2 in mouse BMSCs via the WNT signaling pathway can induce the differentiation of BMSCs into cardiomyocytes more effectively [46].

BMSCs can be differentiated not only into cardiomyocyte-like cells but also into endothelial cells, which contribute to the regeneration of the vasculature after MI. For example, it has been demonstrated that atherogenic cytokines regulate VEGF-A-induced differentiation of BMSCs into endothelial cells in vitro [47].

## Problems and prospects

Although transplantation of BMSCs has brought a new option for MI treatment, there are still significant problems to be solved. Because of the lack of a specific marker on the surface of BMSCs, exclusive identification can only be made based on cell shape. Unfortunately, the cellular viability of transplanted BMSCs has not been high because of the lack of blood supply, poor nourishment of transplanted cells, and the generation of free radicals [48]. Spontaneous malignant transformation may represent a biohazard in long-term expansion of human BMSCs in vitro [49]. Furthermore, implanted BMSCs sometimes fail to metabolically stabilize or recover electromechanical function in rat infarcted hearts [50]. A few literature studies have reported anti-fibrotic, immunomodulatory, and anti-inflammatory effects of BMSCs after MI, as we have already documented. Moreover, current research has remained at the basal level, with little clinical research about the treatment of MI by transplantation of BMSCs in human trials. Therefore, greater efforts should be made to investigate the application of BMSCs for the regeneration of cardiomyocytes after MI. At present, the most important goal is to improve the survival rate of BMSCs after infarction transplantation, which will form the basis for solutions to the remaining problems. A large number of clinical trials should be implemented to confirm the optimal dose and route of administration of BMSCs in the treatment of MI patients.

## Conclusions

BMSCs can serve as ideal seed cells in cardiovascular disease therapy because of their capacity for multidirectional differentiation, easy acquisition, rapid proliferation, low immunogenicity, and high portability. Furthermore, transplanted BMSCs can reduce apoptosis, migrate to infarcted myocardial tissues, inhibit fibrosis and inflammation, promote neovascularization, and differentiate into cardiomyocyte-like cells which contribute to the repair of cardiomyocytes. Despite these advantages of BMSCs in the treatment of MI, the most important problem we must now address is the reduced viability of transplanted BMSCs due to inadequate blood supply, poor nourishment of cells, and generation of free radicals. In clinical trials, the feasibility and safety of BMSC therapy have been tested, but the optimal dose and route of administration of BMSCs should be investigated in the treatment of MI.

## Abbreviations

bFGF: Basic fibroblast growth factor
BMSC: Bone marrow mesenchymal stem cell
CXCR4: CXC chemokine receptor 4
Epac: Exchange protein directly activated by cAMP
HGF: Hepatocyte growth factor
HIF-1α: Hypoxia-inducible factor 1α
IGF: Insulin-like growth factor
IL-6: Interleukin-6
LV: Left ventricular
MHC: Major histocompatibility complex
MI: Myocardial infarction
miR: MicroRNA
MSC: Mesenchymal stem cell
Nrf2: Nuclear factor (erythroid-derived 2)-like 2
PGE-2: Prostaglandin E-2
SDF-1: Stromal cell derived factor 1
TNF: Tumor necrosis factor
VEGF: Vascular endothelial growth factor
